# A Framework for Aligning Data from Multiple Institutions to Conduct Meaningful Analytics

**DOI:** 10.5334/egems.195

**Published:** 2017-12-15

**Authors:** Jay Knowlton, Tom Belnap, Bonnie Patelesio, Elisa L. Priest, Friedrich von Recklinghausen, Andreas H. Taenzer

**Affiliations:** 1 High Value Healthcare Collaborative, US; 2 Intermountain Healthcare, US; 3 Hawai’i Pacific Health, US; 4 Baylor Scott and White Health, US; 5 Dartmouth-Hitchcock Health, US; 6 Dartmouth-Hitchcock Health, The Dartmouth Institute, US

**Keywords:** Methods, Informatics, Learning Health System

## Abstract

**Introduction:**

Health systems can be supported by collaborative networks focused on data sharing and comparative analytics to identify and rapidly disseminate promising care practices. Standardized data collection, quality assessment, and cleansing is a necessary process to facilitate meaningful analytics for operations, quality improvement, and research. We developed a framework for aligning data from health care delivery systems using the High Value Healthcare Collaborative central registry.

**Framework:**

The centralized data registry model allows for multiple layers of data quality assessment. Our framework uses an iterative approach, starting with clear specifications, maintaining ongoing dialogue with diverse stakeholders, and regular checkpoints to assess data conformance, completeness, and plausibility.

**Lessons Learned:**

We found that an iterative communication process is critical for a central registry to ensure: 1) clarity of data specifications, 2) appropriate data quality, and 3) thorough understanding of data source, purpose, and context. Engaging teams from all participating institutions and incorporating diverse stakeholders of clinicians, information technologists, data analysts, operations managers, and health services researchers in all decision making processes supports development of high quality datasets for comparative analytics across multiple institutions.

**Conclusion:**

A standard data specification and submission process alone does not guarantee aligned data for a collaborative registry. Implementing an iterative data quality improvement framework with extensive communication proved to be effective for aligning data from multiple institutions to support meaningful analytics.

## Introduction

Health care delivery systems have a long history of using their own internal data to assess and improve performance. In recent years, an increasing number of systems are looking beyond their own population to assess how they compare to other delivery systems. This often occurs by contributing data for quality measure benchmarking and to allow participating organizations to understand how their own level of care fits within a broader health care system. The High Value Healthcare Collaborative (HVHC) is a group of twelve leading health care delivery systems and The Dartmouth Institute for Health Policy and Clinical Practice (TDI); these organizations are the Collaborative “Members” from across the United States and are focused on improving health care value through data and collaboration. Unlike other data-based approaches to benchmarking, HVHC uses data as the basis for an environment focused on shared learning and collaboration on quality improvement projects.

Nationally, there are multiple efforts to standardize data for quality improvement and research. These networks take the approach of either a centralized data repository, where all data is submitted and stored at a central location, or a distributed network, where data remains at the participating organizations. Some newer initiatives, such as The Patient Centered Outcomes Research Institute Network (PCORnet) [1], use a combination of distributed and centralized models throughout the network. Others, such as the Observational Medical Outcomes Partnership (OMOP) [2] and the Health Care Systems Research Network (HCSRN) [3], use distributed models. HVHC is unique because it is a large scale, not for profit, centralized model focused on rapidly improving health care value.

HVHC uses a centralized data model housed at their Program Management Office (PMO) within TDI. In addition to sharing data, HVHC Members participate in collaborative quality improvement efforts to learn from each other and rapidly change practice across delivery systems. In order to make this centralized approach to data sharing successful within the context of HVHC, an infrastructure has been developed, key stakeholders from Member organizations have designed data collection methods, and clinical teams have set priorities for HVHC projects. Standardization has been key to sharing meaningful data and as part of that process there has been a considerable learning curve to ensure we are sharing comparable data. This has resulted in structured submission, validation, and analytic processes designed to inform improvement projects and clinical practice.

## Data Submission Process Framework

We developed a data submission framework that relies on shared input, regular full-team communications, and ongoing conversation between the coordinating staff at the PMO and the data operations staff at each submitting Member organization. This model began with centralized Institutional Review Board (IRB) approval (with local supplementation as-needed), the development of a standardized data specification, which was led by a group of clinical, operational, and research-oriented professionals (collectively, the “Project Team”) with input from multi-disciplinary stakeholder groups across the Collaborative. The specifications were designed to align with national standards (e.g., CMS measures) to streamline the data collection process for Member organizations. Prior to final approval, a select number of sites submitted pilot data submissions and any learnings were incorporated into the final specification.

The final data specification was released to the broader Collaborative stakeholders during a scheduled webinar where all submitting Member organizations’ representatives asked questions and the Project Team provided context to the structured format, variable definitions, and requested units of measurement. A “Frequently Asked Questions” (FAQ) document was developed and updated throughout the project period. Regularly scheduled meetings of the broader Collaborative stakeholders allowed for ongoing dialogue, including specification version releases. The most current version of the specification and FAQ document were made available to all stakeholders via a shared web portal.

**Figure 1:**
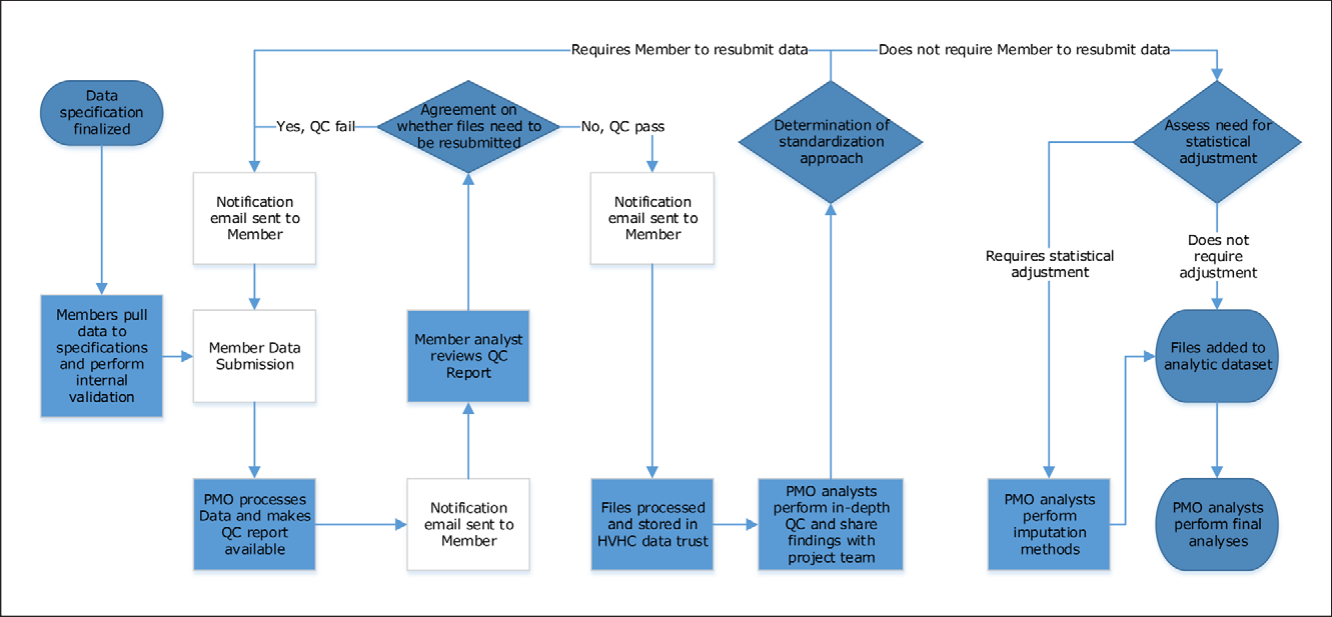
HVHC Data Submission Quality Management Framework.

The coordinating staff at the PMO cataloged lessons learned from each submitting Member organizations’ data files, including noted limitations in data availability, organization-specific approaches to meeting cohort definitions, certain Members’ security department restrictions on sharing particular variables (e.g., payer product line and segment), and resource bandwidth limitations impacting data submission timelines. This documentation was sourced from email communication, web meetings, and in-person discussions. As shown in the Figure 1 framework, the process includes multiple check-points assessing data quality and regular communication between the PMO staff and representatives of Member organizations.

## Sepsis Improvement Work

HVHC designated sepsis care improvement as a priority in 2012 and HVHC Members began collecting and submitting data on a quarterly basis in 2013. The first phase (“Phase I”) of sepsis work focused on improving compliance of a “three hour bundle” of care processes and was supported by an Innovation Award from the Center for Medicare and Medicaid Innovation (CMMI) [4]. This protocol, if completed within the first three hours following suspicion of sepsis is shown to result in improved outcomes [5]. To facilitate standardized data collection about the patients and processes, HVHC developed a sepsis data specification that outlined table structure, variable naming, formats, and definitions. During Phase I, Members performed data quality assessments before submission to the PMO. At the PMO, data quality checks initially focused on: 1) verification of conformance to the specification document and 2) ad-hoc verification of completeness of the data.

The PMO used the submitted data to determine baseline bundle compliance rates, other sepsis-related metrics (e.g., length of stay, mortality), and to assess improvement over time. Results were presented to HVHC Members and discussed during Project Team meetings, which uncovered that the data collection and quality assessment process varied by organization. For example, multiple sites used a combination of manual and electronically abstracted data to collect the needed elements, while other sites used either an all-manual or all-electronic approach. These variations impacted the process of care metrics, required analytical adjustments, and made some analyses more complex to interpret.

After more than a year of experience with Phase I sepsis data submissions, the Project Team determined that changing evidence in sepsis care required modifications to the sepsis metrics and data specifications [6,7,8]. Additionally, the onboarding of new HVHC Members along with a new strategic focus on rapid dissemination and implementation of promising care practices (“best practices”) prompted HVHC to move the sepsis project to a new, second phase. Phase II work was supported by a grant from the Laura and John Arnold Foundation to leverage HVHC’s investment in collaborative sepsis care improvement as a pilot for the HVHC framework of rapid, broad-scale dissemination and implementation of best practices.

## Key Lessons Learned

With Phase II of the sepsis work, the Project Team aimed to improve the data submission process. This required modifications to the existing data specification, expansion of data quality assessment processes at the PMO, and extensive communication and documentation to Members. These changes required an iterative approach and close communication between the Project Team, PMO staff, and HVHC Members.

### Data Specifications

Based on new evidence in sepsis care [6,7,8], Phase II expanded the required data elements to include outcome data as well as process data. For example, we added Intensive Care Unit Length of Stay (ICU LOS) in hours as an outcome. The sepsis specification did not include the source of the data extraction, but rather a defined table structure, data element definition, and format. Further, the table structure was redesigned to overcome challenges in merging the HVHC Member data with available CMS Medicare claims data. To facilitate this, Members were able to leverage the HVHC Master Collaboration Agreement (MCA), which outlines contractual approval for submission of direct patient identifiers; further, the Dartmouth College IRB serves as the HVHC IRB of record in addition to local IRB approvals as-needed by some Member organizations’ institutional requirements. To comply with federal data security rules, the table structure was designed so that direct patient identifiers could be easily segregated when received and stored by the PMO. Each of these changes added a layer of complexity for both the Member organizations and the PMO. For example, one Member required an additional IRB and security review by their own institutions’ entities and could not submit all required data elements until midway through Phase II. This impacted the ability of PMO staff to adequately assess data quality of the aggregate dataset used for comparative reporting.

Based on feedback from the HVHC Members, the specification documentation was also improved by adding more detailed documentation about variable definitions where needed, including development of the FAQ document. In addition, some variable definitions were modified to align with changes in CMS regulations that began to require reporting for the Sepsis Core measure SEP-1 [9].

### Data Quality Assessment

Data quality assessment during Phase II expanded previous programmed checks to include 1) verification and validation of conformance to the specification document; 2) verification and validation of completeness of the newly added data; and 3) additional validation checks to ensure values fall within an appropriate range and are plausible, as shown in Table 1. This expanded data quality assessment follows the three categories outlined in the developing framework for secondary use of electronic health record data [10].

**Table 1:** Data quality assessment during Phase I and Phase II of HVHC sepsis care improvement work.

	HVHC Sepsis Data Quality Assessment	

	Phase I	Phase II

**File Structure**	All fields are found in the specified order using the specified naming conventions	All fields are found in the specified order using the specified naming conventions
**Data Completeness**	Ad-hoc distribution analyses of variables in question for each analysis	Programmed logic identifying percent missingness of each required field
**Data Accuracy**	Ad-hoc identification of improper coding when performing downstream analyses	Programmed logic identifying valid values based on specified codes, appropriate dates (e.g., no future dates, birth date prior to death date), and general plausibility (e.g., ICU length of stay no longer than total inpatient length of stay)

**Figure 2:**
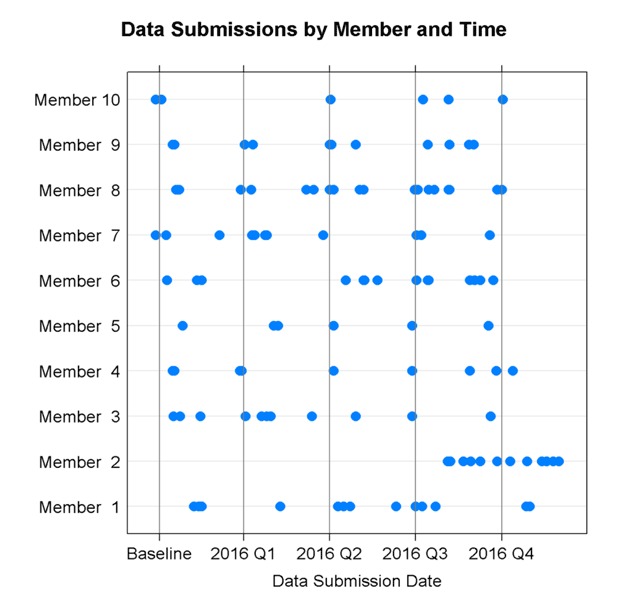
Phase II sepsis data submissions by HVHC Member and submission due date.

With Phase II, the PMO also increased the communication about data quality assessment with HVHC Members; as described in Figure 1, results of each data quality check were provided to the Members through shared web portal and then a phone call was scheduled to discuss the findings. This allowed Members to review the checks independently before meeting with the PMO. After discussing the data quality results, the Members and PMO collaboratively determined whether the Member needed to update and resubmit the data. Each Member organization had at least one – and usually multiple – conversations around data quality with PMO staff, generally resulting in data resubmissions. Figure 2 demonstrates the variability in Members’ ability to submit timely and clean data, and highlights that aggregate data quality was not rapidly improved with the first round of submissions, requiring an iterative improvement process with each data submission. As new data quality issues were identified, new quality checks were added to the programs generating reports available to Members via the web portal.

### Communication

Over time, the PMO’s data quality assessment coupled with extensive communication and work by the HVHC Members resulted in improved data quality. The quality checks identified issues in the data which resulted from errors in programming, formatting, and transmission, or from changes to the Member organizations’ underlying data systems. When possible, the Members corrected these issues. However, the review process also identified a set of known data issues inherent in the data. For example, one Member identified that a change in how the electronic health record stored patient ethnicity resulted in missing values. This could not be corrected until the system was updated several months later. The consistent line of communication allowed the PMO to better understand and document the complex issues faced by each Member and helped the Member organizations improve submissions throughout the course of Phase II. As Kahn et al. (2015) laid out, collecting data on the data source, data capture method, and original purpose of the data is critical [11]. In addition to these recommendations, the PMO also began documenting email and web meeting communications related to Members’ internal processes and resource allocations. A cumulative list of all known data anomalies and limitations for each Member was maintained during the project, which was regularly reviewed with the Project Team as context for decision making related to timelines, specification changes, and desired analytic deliverables.

The more significant quality assessment, communication, and documentation during Phase II helped to uncover several major sources of the observed variation in data quality. Although it was known in Phase I that different HVHC Members were extracting data using a variety of methods (e.g., manual vs. electronic abstraction), Phase II revealed that data originated from different systems as well; these included billing, clinical, or quality reporting systems. For example, one Member used a combination of their quality reporting system (used for regulatory submission of the SEP-1 CMS measure) and administrative data. Another Member had state-specific sepsis reporting requirements with a separate data warehouse for sepsis encounter data, making it difficult to then merge all HVHC-requested data elements into the standard Collaborative specification. These examples led to inconsistencies in fields such as diagnosis codes associated with patient encounters, each of which were only uncovered after communication by the PMO and research by the Member organization (e.g., the Member with state-specific sepsis reporting has a sepsis data warehouse where every encounter has been manually validated, but it does not include structured data fields for diagnosis codes so they were omitted submission to HVHC, making benchmarking by diagnosis code impossible for that Member).

Communication and documentation also revealed variations in interpretation of data definitions. For example, identification of sepsis recognition time varied in method and definition. Members used prospective data collection by clinicians during the sepsis event, retrospective extraction from the medical record by nurse abstractors, or electronic abstraction by analytics or information technology staff. Each of these methods resulted in variations in the definition and meaning of the sepsis recognition time; this contributed to complexity when interpreting analyses, but the project team felt strongly that each documented approach was appropriate for the respective Members. Further, ambiguity around appropriate use of certain national standardized codes (e.g., intermediate ICU revenue center codes) can lead to variation by organization. This issue is explored in depth in the accompanying article by von Recklinghausen et al. [12] in this issue and calls into question whether national standardized codes can be relied on to generate standardized data for sophisticated improvement initiatives; additional clarity for data use and measure interpretation proved to be helpful and should be considered when determining data definitions for multi-institutional reporting.

## Standardization Methods

Despite an extensive specifications document, expanded data quality assessment process, and communication with submitting Members, there were still challenges with the data that required unique standardization methods. Assembling a reliable analytic dataset based on submissions from multiple institutions requires understanding institutional differences in data sources and collection methods. HVHC has used the approach of engaging multidisciplinary teams from across the Collaborative to understand norms and engage specific stakeholders at sites with observed data anomalies. The combination of site-specific root cause investigation with broader industry understanding allows researchers (i.e., project teams) to devise a meaningful approach to standardizing data. For example, when on Member’s ICU LOS reported to HVHC appeared notably different than found in CMS claims, the PMO convened a group of analysts, informaticists, billing specialists, and quality improvement professionals at that organization to understand the data sources and workflows contributing to HVHC data reporting; this is explored further in the accompanying article by von Recklinghausen et al. [12].

Once data anomalies have been identified and understood through working with project teams and submitting Members, steps are taken to correct any errors. Whenever possible, the PMO prefers that a Member resubmit data to address areas of concern. Data submissions were structured so that when the most recent quarter’s data were transmitted, the preceding quarters were also included; this cumulative structure allowed for a full data refresh with each submission and helped to minimize the need for repeated submissions of a single quarter’s data.

Phase II of the sepsis work provided many instances where Members were asked to resubmit data; an example of this (LOS unit of measurement) is shown in Table 2. Rather than convert the unit of measurement at the PMO, Members were expected to follow their internal conversion and rounding logic for submission to HVHC. This method was preferred due to concerns about losing data specificity, and in order to help ensure Members update coding for future data submissions.

**Table 2:** Institutions’ misalingment with LOS specifications.

LOS Reporting Variant	Number of Institutions	Time to Alignment with Specification

Reported in hours	6	N/A
Reported in days rounded to nearest integer	1	3 months (corrected with next submission)
Reported in days rounded to nearest integer; any value <1 rounds UP	1	6 months (failed to correct with next submission; corrected with subsequent submission, adjusted by PMO in the interim)
Reported in days rounded UP to nearest integer	1	3 months (corrected with next submission)

In cases where it was not possible for Members to submit updated data, the multidisciplinary Project Team developed statistical approaches based on available data and site-specific context. Examples of this include imputation of data points for Members missing one or more quarters’ worth of encounter data, Members missing encounters for patients who were never transferred to the intensive care unit (ICU), and Members with identified discrepancies between clinical data submitted to HVHC and data observed in CMS claims. Each of these examples required significant root cause analysis by PMO staff, the Project Team, and Member representatives before determining the appropriate adjustment methodology to meet the analytic needs of Phase II deliverables. Further details explaining the analytic approaches used to addressing data inconsistencies are reviewed in the accompanying paper by Welch et al. [13] within this issue.

## Conclusion

Despite having a standard specification and submission process, the centralized quality control process found discrepancies in submitted data. Those discrepancies were uncovered, communicated, and, when possible, mitigated by the additional validation work performed by the HVHC PMO after Members performed internal validation. This serves as a good example of how, despite rigorous standardization efforts, differences may persist due to underlying differences in workflow, data capture, and electronic health record capabilities. Without careful protocols in place and a structured process that allowed appropriate time to assess, validate, and investigate data quality, many of these differences would have gone undetected.

These processes were effective in developing a well-aligned dataset from multiple health care delivery systems, but they were not able to overcome all data issues that were encountered. It became important for the PMO to identify the use cases for the data and determine how clean the data need to be to carry out those analyses, also known as “fitness for use”. In many cases the data do not need to be perfect (i.e., entirely conform to the data specification) to detect the level of differences required to achieve the project’s analytic goals. However, to reduce bias in analyses, it was still important for the team to identify where variation occurred, why the variation existed, and how that variation was likely to impact analytic deliverables.

It is important to note that this approach has several limitations. There likely are other data issues that remained undetected, and the issues that were identified could not be mitigated in all circumstances; at the time of analysis significant differences still existed. Like most projects, there were time and resource availability limitations preventing more comprehensive standardization work from being completed. As a result it was necessary to determine what level of standardization was acceptable for the needs of the project (e.g., allowing sites flexibility in for defining time zero based on operational processes in place at respective organizations, under conditional approval by the project team for conformation to CMS SEP-1 guidelines). Despite these limitations, at the time of analysis the data were significantly better fit for the HVHC-intended use than they would have been otherwise. With our lessons learned, we attribute this iterative improvement to three framework principles that we were able to enhance over time: 1) clear data specifications developed with broad stakeholder input; 2) robust data quality assessment using an iterative improvement framework at the central registry; and 3) extensive communication by the PMO to understand context for the data being used. These principles proved to be effective for aligning data from multiple institutions to conduct meaningful analytics.
